# Factors Predicting Early Major Adverse Events in the Intensive Care Unit After Successful Cardiac Surgery for Congenital Heart Disease in Full-Term Neonates

**DOI:** 10.21470/1678-9741-2022-0442

**Published:** 2023-07-18

**Authors:** Dilek Yavuzcan Oztürk, Erkut Oztürk, Hatice Dilek Ozcanoglu, Ibrahim Cansaran Tanıdır, Merih Çetinkaya, Ali Can Hatemi

**Affiliations:** 1 Department of Neonatology, Istanbul Saglik Bilimleri University Basaksehir Cam and Sakura Hospital, Istanbul, Turkey; 2 Department of Pediatric Cardiology, Istanbul Saglik Bilimleri University Basaksehir Cam and Sakura Hospital, Istanbul, Turkey; 3 Department of Anaesthesiology and Reanimation, Istanbul Saglik Bilimleri University Basaksehir Cam and Sakura Hospital, Istanbul, Turkey; 4 Department of Pediatric Cardiovascular Surgery, Istanbul Saglik Bilimleri University Basaksehir Cam and Sakura Hospital, Istanbul, Turkey

**Keywords:** Newborn Infant, Risk Factors, Cardiac Surgery, Lactates, Reoperation, Area Under Curve, Intensive Care Units, Confidence Intervals

## Abstract

**Objective:**

In this study, we aimed to evaluate the factors affecting major adverse event (MAE) development after full-term neonatal cardiac surgery.

**Methods:**

This study was conducted retrospectively on newborns who underwent congenital heart surgery between June 1, 2020, and June 1, 2022. MAE was defined as the presence of at least one of the following: cardiac arrest, unplanned reoperation, emergency chest opening, admission to the advanced life support system, and death. The role of blood lactate level, vasoactive inotropic score (VIS), and cerebral near-infrared spectroscopy (NIRS) changes in predicting MAE was investigated.

**Results:**

A total of 240 patients (50% male) were operated during the study period. The median age of patients was seven days (interquartile range 3-10 days). MAE was detected in 19.5% of the cases. Peak blood lactate levels >7 mmol/liter (area under the curve [AUC] 0.72, 95% confidence interval [CI] [0.62-0.82], P<0.001, sensitivity 76%, specificity 82%, positive predictive value [PPV] 88%) was an independent risk factor for MAE (odds ratio [OR] 2.7 [95% CI 1.3-6]). More than 30% change in NIRS value during the operative period (AUC 0.84, 95% CI [0.80-0.88], P<0.001, sensitivity 65%, specificity 85%, PPV 90%) was a strong predictor of MAE. VIS > 10 was an independent risk factor (AUC 0.75, 95% CI [0.70-0.84], P<0.001, sensitivity 86%, specificity 80%, PPV 84%) and strongly predicted MAE (OR 1.4 [95% CI 0.9-5]).

**Conclusion:**

Cerebral NIRS changes > 30%, high blood lactate levels, and VIS score within the 48 hours may help to predict the development of MAE in the postoperative period.

## INTRODUCTION

**Table:** 

Abbreviations, Acronyms & Symbols				
AUC	= Area under the curve		NIRS	= Near-infrared spectroscopy
CHD	= Congenital heart diseases		OR	= Odds ratio
CI	= Confidence interval		PICU	= Pediatric intensive care unit
CPB	= Cardiopulmonary bypass		PPV	= Positive predictive value
ECMO	= Extracorporeal membrane oxygenation		ROC	= Receiver operating characteristic
ICU IQR	= Intensive care unit = Interquartile range		STAT	= The Society of Thoracic Surgeons-European Association for Cardio-Thoracic Surgery
LCOS	= Low cardiac output syndrome		VIS	= Vasoactive inotropic score
MAE	= Major adverse event			

Congenital heart diseases (CHD) are heterogeneous disorders including various pathologies and subgroups. The prevalence of CHD is 0.6-1%, and 25% of patients have clinically significant diseases requiring surgery within the first year of life. A timely and accurate diagnosis followed by appropriate treatment is essential to increase survival^[1]^.

Neonates with critical CHD requiring surgery with cardiopulmonary bypass (CPB) are at high risk for morbidity and mortality. The postoperative 30-day mortality rates following complex procedures range from 5% to 10%, which is relatively high compared to any other age group. Several neonatal-specific risk factors have been identified for adverse outcomes after cardiac surgery, such as gestational age, low birth weight, the complexity of the surgical procedure, and having single-ventricle physiology. A major adverse event (MAE) can be seen in newborns and other age groups and adversely affect the operation success^[2]^. MAE was defined as the presence of at least one of the following: cardiac arrest (with or without extracorporeal life support), unplanned reoperation emergency, chest opening, or death^[3-5]^. Identifying patients at risk for MAE is challenging, but it could help physicians and nurses to monitor and allocate more resources to specific patients to prevent or rapidly address and treat a MAE.

The complexity of CHD and various complications following operation cause severe consequences in the management and decision-making of newborns. Physical examination, non-invasive/invasive hemodynamic monitorization, near-infrared spectroscopy (NIRS), and cerebral oxygen levels, scoring systems, and blood lactate level are used in combination to overcome these difficulties^[2-4]^. Although blood lactate level and cerebral NIRS are used as indirect markers showing tissue hypoxia, the contribution of these methods in predicting MAE after neonatal cardiac surgery is questionable, and there are limited studies on this subject.

This study investigated the frequency of MAE in patients who underwent full-term neonatal cardiac surgery in our cardiac surgery center. In addition, the contributions of various parameters to MAE and its predictors were investigated.

## METHODS

This study was conducted retrospectively on newborns who underwent surgery for CHD between June 1, 2020, and June 1, 2022. Premature infants, patients with known neurological diseases, intraoperative deaths, and surgeries without CPB were excluded from the study. The study was conducted in accordance with the Helsinki Declaration after local ethics committee approval (2022/95).

A study form was created for each case. The study form was divided into three sub-headings; *preoperative data* (demographic characteristics, cardiac pathology, echocardiographic data), *operative data* (duration of operation, CPB time), surgical risk scores (The Society of Thoracic Surgeons Congenital Heart Surgery Database [or STS-CHSD] and the European Association for Cardio-Thoracic Surgery (or EACTS) Risk Adjustment for Congenital Heart Surgery^[6]^, cerebral NIRS, blood lactate levels, mixed venous oxygen saturation), and postoperative data (extubation time, duration of intensive care and hospital stay, mortality, vasoactive inotropic score [VIS], blood gas analysis, MAE).

Our clinic’s routine perioperative anesthesia protocol was used for surgery. In accordance with this protocol, 0.1 mg/kg midazolam, 10 micrograms/kg/fentanyl, and 0.6 mg/kg rocuronium were administered for induction. Remifentanil 0.2 micrograms/kg/min, rocuronium 5 micrograms/kg/min, and sevoflurane 1-1.2 minimum alveolar concentration was used for anesthesia maintenance.

A four-channel trend monitor (Somanetics 5100B, Troy, Michigan, United States of America) was used for cerebral monitoring. The NIRS sensor was placed on the frontal region for children. Baseline NIRS values were recorded as the first measured values after admission to the intensive care unit (ICU), and cerebral oxygenation changes were evaluated. Following this initial measurement, changes over 30%, 20%, and 10% within the first 48 hours were recorded.

Blood sample for lactate level was collected from the arterial cannula inserted during surgery. Blood lactate levels are routinely collected on cardiac ICU admission and frequently thereafter (*i.e.*, six, 12, 24, and 48 hours, and more often if clinically indicated) during the postoperative period^[7]^. Blood lactate levels (mmol/l) were defined according to the following time intervals: the value on admission to the cardiac ICU following transfer from the operating room; the delta level from cardiac ICU admission to 12 hours; and the peak value within the initial 48 postoperative hours in the cardiac ICU or earlier if the defined outcome occurred prior to 48 hours^[7]^. The blood sample venous oxygen saturation was measured simultaneously with NIRS.

VIS values were calculated for each patient by a standard formula for the first 48 postoperative hours (every hour), and the maximum score was recorded^[8]^. VIS: dopamine dose (µg/kg/min) + dobutamine dose (µg/kg/min) + 100 × epinephrine dose (µg/kg/min)] + 10 × milrinone dose (µg/kg/min) + 10,000 × vasopressin dose (Units/kg/min) + 100 × norepinephrine dose (µg/kg/min). Inotropic dose adjustment was based on low cardiac output syndrome (LCOS) findings. LCOS was defined by clinical changes such as altered level of consciousness, alteration of skin appearance, cold extremities, weak pulse, and capillary refill time > 2 seconds.

Intraoperative epicardial and postoperative transthoracic echocardiography within 24 hours of the surgery, usually before extubation in the ICU, were performed routinely in all patients.

Our primary outcome was the occurrence of a MAE within 48 hours after surgery - at least one of the following: cardiac arrest, unplanned reoperation, emergency chest reopening, admission to advanced life support system, or death. Unplanned reoperation was defined as the need for an additional, unanticipated surgical procedure or revision as a result of a significant postoperative residual lesion (technical scores are those of class 3). The technical performance score is a tool that was developed to grade the adequacy of surgical repair as “Class 3: major residua or reintervention for major residua during index hospitalization, inadequate’ based on echocardiographic and clinical criteria”. Emergency chest reopening was defined as the need of sternum opening for exploration, bleeding control, or to alleviate pressure on the mediastinum.

### Statistical Analysis

Statistical analysis was performed using IBM Corp. Released 2012, IBM SPSS Statistics for Windows, version 21.0, Armonk, NY: IBM Corp. Results for continuous variables with normal distribution were presented as mean (standard deviation), and non-normally distributed data were reported as median (interquartile range [IQR]). Categorical variables were presented as numbers and percentages. Demographic characteristics and perioperative variables were compared with Mann-Whitney U and chi-square tests. The effect of parameters in predicting MAE was assessed by the receiver operating characteristic (ROC) curve. Significance variables were included in the multivariate logistic regression model, and odds ratio (OR) was calculated. *P*<0.05 was considered statistically significant.

## RESULTS

In this period, 310 newborns were operated. Premature infants (n=35), patients with known diseases (n=6), intraoperative deaths (n=3), and surgeries without CPB (n=26) were excluded from the study; 240 full-term neonates (50% male) were included in the study.

The median age of patients was seven days (IQR 3-10 days). The demographic and clinical characteristics of all patients were summarized in Table 1.

**Table 1:** Demographics and patient characteristics.

Variables	Median (IQR) or n%
n	240
Age at surgery (days)	7 (3-10)
Weight at surgery (kg)	3 (2.8-3.2)
Body surface area (m^2^)	0.20 (0.18-0.22)
Male	120 (50)
STAT category	
1-2-3	24 (10)
4	156 (65)
5	60 (25)
Syndrome	16 (6.5)
Emergency procedure	96 (40)
Main diagnosis	
Aortic arch hypoplasia with or without coarctation of the aorta	40 (16)
Hypoplastic left heart syndrome	34 (14)
Pulmonary atresia (intact ventricular septum or with ventricular septal defect)	27 (11)
Total anomalous pulmonary venous return	32 (13)
Transposition of the great arteries (simple or complex)	65 (27)
Truncus arteriosus	12 (5)
Other	30 (8)
Physiology	
Single-ventricle	60 (25)
Biventricular	180 (75)

IQR=interquartile range; STAT=The Society of Thoracic Surgeons-European Association for Cardio-Thoracic Surgery

MAE was detected in 19.5% of the cases. Of the 47 patients who experienced MAE, 30 died (12.5%), 18 had cardiac arrest (7.5%), four (1.6%) had residual hemodynamic lesion, four (1.6%) had sternum reopening, and 10 (4%) required extracorporeal membrane oxygenation support (Table 2).

**Table 2:** Post-cardiac surgery major adverse event outcomes.

Outcomes	n (%)
Major adverse event	
Yes	47 (19.5)
No	193 (81.5)
Cardiac arrest within 48 hours	
Yes	18 (7.5)
No	222 (92.5)
ECMO within 48 hours	
Yes	10 (4)
No	230 (96)
Intra-hospital mortality within 30 days	
Yes	30 (12.5)
No	210 (87.5)
Residual hemodynamic defect	
Yes	4 (1.6)
No	236 (88.4)
Chest open within 48 hours	
Yes	4 (1.6)
No	236 (88.4)
Postoperative time to composite major adverse event	
0 to < 6 hours	19 (40)
6 to < 12 hours	12 (25)
12 to < 24 hours	9 (20)
24 to ≤ 48 hours	7 (15)

ECMO=extracorporeal membrane oxygenation

LCOS was developed in 42 patients. The effect of various parameters in the development of MAE were shown in Table 3. Neonates who sustained the composite outcome had significantly higher lactate levels on cardiac ICU admission and peak lactate levels within 48 hours compared to those without the outcome with medians of 5.9 mmol/l (IQR: 4, 7.7) *vs.* 4.2 mmol/l (IQR: 3, 6.8) (*P*=0.010), and 6.8 mmol/l (IQR: 5, 8.9) *vs.* 4.7 mmol/l (IQR: 3.3, 6.1) (*P*=0.001). The patient group with MAE had more single-ventricle physiology (57% *vs.* 17%), longer median CPB duration (115 min *vs.* 95 min), higher median peak VIS (7 *vs.* 15), and higher 30% change in cerebral NIRS (44% *vs.* 2%).

**Table 3:** Evaluation of variables according to the presence of major adverse events (MAEs).

Variable	MAE (+)	MAE (-)	*P*-value
Age at surgery, days	5 (3-7)	7 (5-10)	NS
Male	24 (51)	96 (49)	NS
Weight at surgery, kg	2.9 (2.7-3.2)	3.1 (2.9-3.4)	NS
Syndrome	6 (12)	10 (5.1)	NS
CPB duration, minutes	115 (100-135)	95 (70-120)	0.002
STAT 4-5	47 (100)	169 (88)	NS
Single-ventricle	27 (57)	33 (17)	0.003
Lactate			
Lactate on ICU arrival (mmol/l)	5.9 (4-7.7)	4.2 (3-6.8)	0.010
Lactate on 24 hours (mmol/l)	3.2 (2.5-4)	2.1 (1.8-2.5)	NS
Lactate on 48 hours (mmol/l)	3.4 (3-4.4)	2 (1.5-2.5)	NS
Peak lactate levels within 48 hours (mmol/l)	6.8 (5-8.9)	4.7 (3.3-6.1)	0.001
Delta lactate from ICU arrival to 12 hours (mmol/l)	0.4 (-1.5-2)	-0.9 (-2-0.4)	0.005
Vasoactive inotropic score (VIS)			
VIS on PICU arrival	10 (7-12)	7 (5-10)	NS
VIS on 24 hours	7 (5-10)	5 (3-7)	NS
VIS on 48 hours	7 (5-10)	5 (3-7)	NS
Peak VIS within 48 hours	15 (10-18)	7 (5-10)	0.001
Delta VIS from ICU arrival to 12 hours	5 (3-7)	2 (1-3)	NS
Cerebral NIRS from ICU arrival to 48 hours			
≥ 30%	21 (44)	4 (2)	0.001
≥ 20%	27 (57)	38 (20)	NS
≥ 10%	41 (89)	104 (54)	NS

Median (interquartile range) or n (%). CPB=cardiopulmonary bypass; ICU=intensive care unit; NIRS=near-infrared spectroscopy; PICU=pediatric intensive care unit; STAT=The Society of Thoracic Surgeons-European Association for Cardio-Thoracic Surgery; VIS=vasoactive inotropic score

**Fig. 1:**
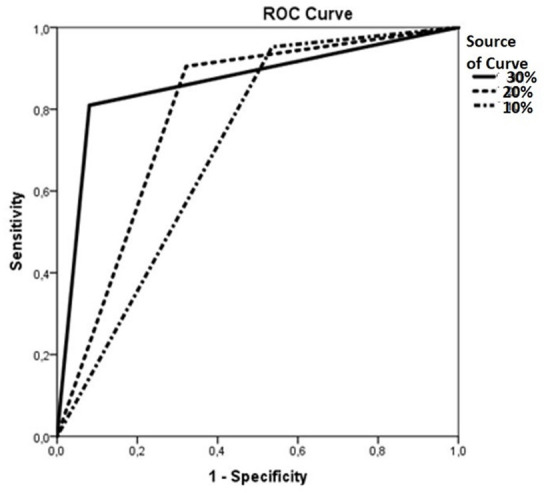
The role of near-infrared spectroscopy changes in predicting major adverse events; ROC=receiver operating characteristic.

Prediction point assessments were performed according to the percentage change in the NIRS values. ROC analysis for the predictive value of 30%, 20%, and 10% change in the cerebral NIRS value during the first 48 hours in estimating MAE was shown in Figure 1. More than 30% change in NIRS value during the operative period (area under the curve [AUC] 0.84, 95% confidence interval [CI] (0.80-0.88), *P*<0.001, sensitivity 65%, specificity 85%, positive predictive value [PPV] 90%) estimated MAE as an independent risk factor; 20% and 10% changes in NIRS value did not show statistical significance (*P*>0.05).

**Fig. 2:**
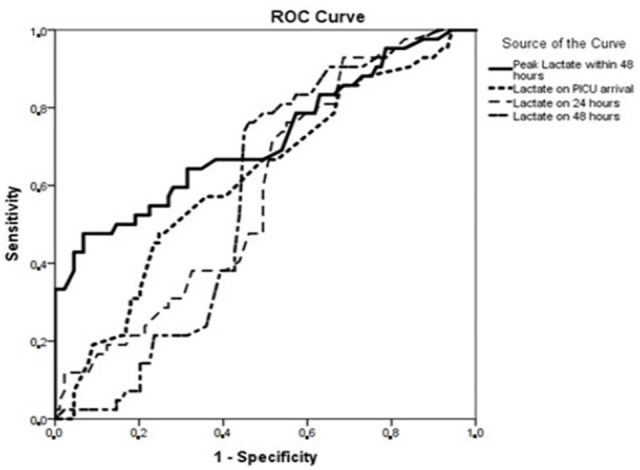
The role of lactate changes in predicting major adverse event. PICU=pediatric intensive care unit; ROC=receiver operating characteristic.

The results of ROC analysis of blood lactate levels in predicting MAE are summarized in Figure 2. Peak blood lactate levels > 7 mmol/liter (AUC 0.72, 95% CI [0.62-0.82], *P*<0.001, sensitivity 76%, specificity 82%, PPV 88%) was an independent risk factor for MAE (OR 2.7 [95% CI 1.3-6]). Also blood lactate level change > 1.8 mmol/liter within first 12 hours was another independent risk factor (OR 6 [95% CI 2.3-9]) and strongly predicted MAE (AUC 0.80, 95% CI [0.70-0.90], *P*<0.001, sensitivity 84%, specificity 90%, PPV 91%).

**Fig. 3:**
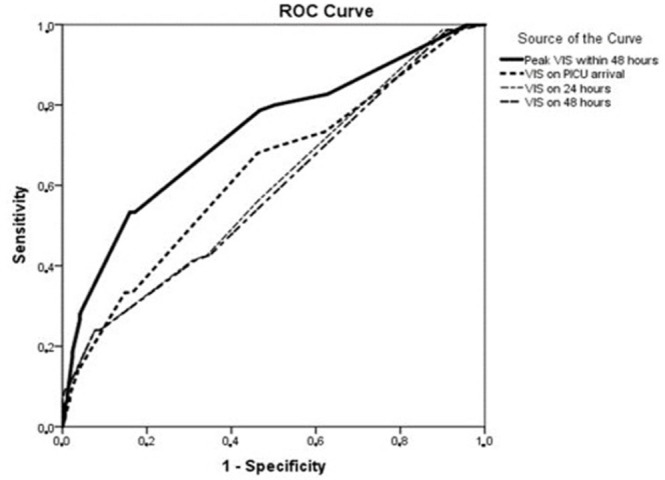
The role of vasoactive inotropic score (VIS) changes in predicting major adverse events. PICU=pediatric intensive care unit; ROC=receiver operating characteristic.

The results of ROC analysis of VIS parameters in predicting the MAE are summarized in Figure 3. VIS > 10 was an independent risk factor (AUC 0.75, 95% CI [0.70-0.84], *P*<0.001, sensitivity 86%, specificity 80%, PPV 84%) and strongly predicted MAE (OR 1.4 [95% CI 0.9-5]).

Multivariate regression analysis was performed for all variables that were found to be significant by univariate analysis (*P*<0.05). Other than NIRS, lactate, and VIS score changes single-ventricle physiology (*vs.* biventricular) (OR 3.2, 95% CI 2-8.6, *P*=0.01) and CPB duration > 110 minutes (OR 1.9, 95% CI 1.1-4.6, *P*=0.04) were found to be independent risk factors (Table 4).

**Table 4:** Independent risk factors causing major adverse events with multivariate logistic regression analysis.

Variables	OR	95% CI	*P*-value
Syndrome	1.1	0.9-3.2	0.120
Cerebral NIRS ≥ 30%	2.4	1-4.5	0.001
Peak blood lactate levels > 7 mmol/liter	2.7	1.3-6	0.001
Delta lactate from ICU arrival to 12 hours (mmol/l)	6	2.3-9	0.001
Peak VIS within 48 hours > 10	1.4	0.9-5	0.001
Single-ventricle physiology	3.2	2-8.6	0.01
CPB time > 110/minutes	1.9	1.1-4.6	0.04

CI=confidence interval; CPB=cardiopulmonary bypass; ICU=intensive care unit; NIRS=near-infrared spectroscopy; OR=odds ratio; VIS=vasoactive inotropic score

## DISCUSSION

This study aimed to evaluate the factors affecting MAE after neonatal cardiac surgery. We found that > 30 % cerebral NIRS change, peak blood lactate level > 7 mmol/l, change in blood lactate level > 1.8 mmol/l within the first 12 hours, and peak VIS value > 10 are valuable predictors for MAE development in the first 48 hours. Our study is one of the limited studies in the literature on this topic.

Various methods such as blood gases, blood lactate level, mixed venous oxygen saturation, and brain perfusion pressure are used to continuously monitor the effect of hemodynamic changes in mortality and morbidity^[9,10]^. Cerebral NIRS monitoring to show microcirculatory alterations gained popularity^[9]^. It allows the evaluation of microcirculation in a non-invasive and continuous manner. In their study, Tanidir et al.^[11]^ suggested that NIRS changes instantly give information about hemodynamic changes. In a newborn series of 75 cases, Aly et al.^[12]^ reported that cerebral NIRS changes during CPB affected survival and long-term neurocognitive development. Modestini et al.^[13]^ stated that basal NIRS values measured immediately after endotracheal intubation were associated with poor postoperative outcomes in their series of 565 cases. Lee et al.^[14]^ suggested that cerebral NIRS values < 51% could cause MAE. Our study found that > 30% change in cerebral NIRS values was an independent risk factor in predicting MAE.

Blood lactate levels could give an idea about the operational success of cardiac surgery in newborns. Increased blood lactate level in the postoperative period is secondary to multifactorial pathophysiological etiologies such as decreased cardiac output, insufficient oxygen transport, increased metabolic need, and capillary leak syndrome. At the same time, the measurement of blood lactate is a practical and reproducible test^[7]^. Although lactate clearance, lactate change in time, mixed venous oxygen saturation, CPB duration, and inflammatory markers are used to predict LCOS development in numerous studies, an ideal method to determine MAE has not been found yet^[2]^. Serial lactate measurement has been suggested to predict MAE in a series of one hundred and twenty-nine cases^[15]^. Schumacher et al.^[16]^ claimed that 0.6 mmol/L/hour decreases in blood lactate levels could predict a favorable prognosis with 90% sensitivity and 84% specificity. In their study involving 432 newborns, Valencia et al.^[7]^ suggested that > 1.5 mmol/liter lactate change within the first 12 hours and single-ventricle physiology could predict MAE, besides early mortality, and morbidity. In our study, 1.8 mmol/liter lactate change increased MAE possibility six times and peak lactate level > 7 mmol/liter increased MAE possibility 2.7 times.

High-dose inotropic requirement’s negative effect on postoperative mortality and morbidity has been shown in different studies. Kulyabin et al.^[17]^ suggested that VIS > 12 independently increases the risk of acute kidney injury and mortality in newborns undergoing arcus aorta operation. Butts et al.^[18]^ stated that VIS could predict early morbidity and mortality in newborn cardiac surgery. In a study involving 119 newborns, Dilli et al.^[19]^ found that high VIS (> 15.5) in the first 72 hours increased mortality five times. In our study, we observed that VIS > 10 in the first 48 hours increased MAE 1.4 times.

In the present study, besides lactate, cerebral NIRS, and VIS score, different factors affecting MAE were also determined. Similar to prior studies, single-ventricle physiology was found to be an independent predictor on univariate analysis of adverse outcomes and MAE post-cardiac surgery^[7]^.

It is known that CPB induces systemic inflammation, leading to capillary leak and hemodynamic disorders. In some studies, no relationship between the duration of CPB and MAEs in newborns was reported^[15,16]^. But on the contrary, in some studies, it was stated that it has a negative effect^[20]^.

Duke et al.^[4]^ investigated predictive factors of MAE in their series including CHD. The MAE incidence was 13.3%, and high blood lactate levels, long CPB duration, CO2 difference, and high base deficit in blood gas in the ICU were associated with MAE. Rocha et al.^[2]^ found MAE incidence of 16%, and mixed venous oxygen saturation/lactate > 5 was a good marker for reduced MAE incidence. In a series of 257 pediatric cardiac surgery patients, Murni et al.^[20]^ found MAE incidence of 19% and mortality of 13%. Cyanotic CHD, CPB duration > 120 minutes, more than two inotrope requirements, and increased lactate > 0.75 mmol/L in the first 24 hours were associated with MAE.

In the present study, the incidence of MAE was 19.5%. Compared to other studies, newborn patient population in our study contributed significantly to the high level of MAE. Cerebral NIRS changes within the postoperative 48 hours, high blood lactate levels, and VIS scores strongly predicted MAE.

If this newborn has an additional single-ventricle physiology and long CPB duration, the probability of MAE will increase significantly (sensitivity 96%, specificity 90%).

### Limitations

The main limitation of the study is that it was conducted on a small number of cases at a single center. Another limitation is that the patients’ pathologies are heterogeneous and have different physiological consequences. Although prematurity is known to be a risk factor for MAE, premature babies were not included in this study.

## CONCLUSION

In conclusion, MAE can be seen at a high rate after neonatal cardiac surgery. Cerebral NIRS changes, high blood lactate levels, single-ventricle physiology, long CPB time, and VIS score in the first 48 hours may help to predict the development of MAE in the postoperative period.
